# COVID-19 vaccine information disorder in Chile: a quantitative analysis of fact-checked articles

**DOI:** 10.3389/fpubh.2025.1399336

**Published:** 2025-06-09

**Authors:** Alexis V. Cruz, Vicente Schulz, Eduardo Arriagada, Claudia A. Montero-Liberona, Alexis M. Kalergis

**Affiliations:** ^1^Millennium Institute on Immunology and Immunotherapy, Santiago, Chile; ^2^Facultad de Comunicaciones, Pontificia Universidad Católica de Chile, Santiago, Chile; ^3^Facultad de Ciencias Biológicas, Pontificia Universidad Católica de Chile, Santiago, Chile; ^4^Departamento de Endocrinología, Escuela de Medicina, Facultad de Medicina, Pontificia Universidad Católica de Chile, Santiago, Chile

**Keywords:** information disorder, vaccination process, COVID-19, fact-checking, vaccines, vaccine hesitancy

## Abstract

**Introduction:**

Previous studies have analyzed information disorder during the COVID-19 pandemic, focusing on common characteristics such as content formats, recurring themes, and information dissemination networks. However, in the Latin American region, there is still a gap in studying the specific characteristics of this phenomenon during the COVID-19 vaccination process, as in the Chilean case. Therefore, this study aims to identify the main characteristics of information disorder circulating in Chile during the COVID-19 vaccination process, considering key topics, actors, and media platforms involved.

**Methods:**

We conducted a quantitative content analysis of a dataset of 140 fact-checking articles related to COVID-19 vaccination, sourced from MalaEspina and FastCheck, from March 2020 to December 2022.

**Results:**

We identified the primary characteristics of information disorders that circulated in Chile concerning the COVID-19 vaccination process. Our main findings indicated that information disorder focused on disinformation intended to cause harm through deception. The primary format used was visual and distributed across various platforms.

**Discussion and conclusion:**

We conclude that studying information disorder in specific topics, such as vaccination, is important to understand the phenomenon better and develop strategies to mitigate its impact on society.

## 1 Introduction

The circulation and propagation of inaccurate information have been a concern in worldwide regions and media outlets (1), especially during the COVID-19 vaccination process (2–6). Previous research has confirmed the existence of information disorder during the COVID-19 vaccination period on several social media platforms (7–10); including Chile (11, 12).

During the COVID-19 pandemic and vaccination rollout, people were exposed to an overwhelming volume of information, much of which was inaccurate or misleading. This excessive flow of content, known as an infodemic, made it increasingly difficult for individuals to distinguish between credible information and misinformation, ultimately shaping their decisions about vaccination (13, 14). Infodemics create a wave of both accurate and inaccurate information, complicating access to clear messages, trustworthy sources, and reliable guidance when people need them most (15, 16). While some information simply generates confusion, other forms can be actively harmful, influencing public perceptions and behaviors that undermine vaccination efforts and public health initiatives (15, 17). Beyond its impact on individuals, the COVID-19 infodemic has had far-reaching societal consequences, eroding trust in health institutions and affecting vaccine confidence and uptake (18, 19).

Despite the global caution call promoted by the World Health Organization (WHO) during the COVID-19 pandemic, societies were inevitably exposed to information disorders. Therefore, while society’s had the urgent need to turn to reliable sources of information (20, 21), fact-checking processes on vaccines inaccurate information emerged as essential (22–24). This was Chile’s case, which remained not sufficiently addressed.

Considering the previous aspect, the main objective of this study is to identify key characteristics of information disorder addressed by fact-checking media within the vaccination process in Chile. This task is of paramount importance not only due to the substantial risks associated with inaccurate information about the COVID-19 vaccination process but also because of its direct impact on vaccine hesitancy (25, 26). Montagni et al. (26) found that individuals categorized as ‘anti-vaccination’ or ‘vaccine hesitant’—as opposed to ‘pro-vaccination’—demonstrate a significantly lower ability to detect false or inaccurate information. Therefore, the objective of this research is relevant because of the fact that 70% of Chileans are unable to distinguish between false and accurate online information (12, 27), which, in turn, has a potential risk on the impact of information disorder, and an opportunity to enhance future communication strategies about inoculation within the country and region (28).

## 2 Literature review

### 2.1 Comprehending vaccine information disorder phenomenon

According to the World Health Organization (WHO), an infodemic is *“an overabundance of information and the rapid spread of misleading or fabricated news, images, and videos”* (19). This information overload intensified during the COVID-19 pandemic significantly shaped public perception and decision-making regarding vaccination (13, 14, 29). For instance, Singh et al. (15) found that the excessive circulation of information across media platforms weakened public trust in vaccines and affected their decision to inoculate, while Ouyang et al. (17) identified that greater exposure to social media content increased vaccine hesitancy as individuals struggled to process the overwhelming volume of both, accurate and inaccurate information. Beyond influencing vaccination decisions, the infodemic also had a psychological toll, as the constant exposure to contradictory and excessive information led to anxiety, confusion, and emotional fatigue, further complicating people’s ability to make informed health choices (14, 30).

The sheer volume of information circulating during the COVID-19 pandemic, as described in the concept of the infodemic, presented a complex challenge. While the term broadly captures the overwhelming influx of information, it is crucial to dissect the nature of this information to understand its precise impact. Simply acknowledging an ‘overabundance’ does not address the varying degrees of accuracy and intent behind the disseminated content. It becomes necessary to differentiate between various forms of information disorder to move beyond the general description of the infodemic and delve into the specific mechanisms that shaped public perception.

The proliferation of information disorder has led to an excessive eagerness to oversimplify the phenomenon under the label of “fake news,” contributing to confusion and a failure to distinguish among the various subtleties of the issue (27, 31–33). Following Wardle and Derakhshan (33), this research conceptualizes “information disorder” as a broad phenomenon of disinformation, misinformation, conspiracy theories, false rumors, and misleading content (33, 34). Wardle and Derakhshan (33) identified three types of information disorder: “Disinformation,” false information created to cause harm; “Misinformation,” false information spread without harmful intent, and “Malinformation,” sharing private, real information with the intent to cause harm.

Previous studies have extensively documented the adverse impact of vaccine information disorders on society (35–38). The correlation between this phenomenon has been confirmed by Montagni et al. (26), who found out that “being ‘anti-vaccination’ or ‘hesitant’, rather than ‘pro-vaccination’, was higher among individuals reporting bad detection of fake news, respectively (…), [and that] the risk of being in ‘hesitant’, rather than ‘pro-vaccination’ was higher among individuals having a bad health literacy score” (26, p. 1). These issues align the WHO decision in 2019 to term “vaccine hesitancy” as a noxious experience and classify it as one of the top ten significant threats to global public health (39).

### 2.2 Vaccination disinformation in media

Globally, it has been demonstrated that social media platforms are the primary channels for disseminating disinformation (12, 27). During the COVID-19 pandemic, the WHO proposed concrete measures for social media companies to address and control false and/or misleading content, such as reporting incorrect information by users themselves (40).

To overcome this threat, the journalistic role performance of fact-checkers has been pivotal (41, 42). Meanwhile, traditional media mostly privileged a locally scoped domain; digital online communication widely opened instant dissemination of information on a broader scale across a diverse range of topics (41, 43–45). Therefore, to regulate information disorders on traditional and social media that could harm citizens by adopting incorrect decisions, fact-checkers news verification task is essential (23, 46). In the case of vaccination processes, it is imperative to scrutinize the phenomenon of information disorder within specific contexts, such as the COVID-19 pandemic.

In the case of vaccine disinformation in the media, there is a lack of studies that analyze this phenomenon. In contrast, a plethora of research has examined the characteristics of information disorder within the context of the COVID-19 pandemic. For example, studies have focused on identifying differences in inaccurate information in several countries, along with components of the type of information disseminated, such as format employed, means of dissemination, primary actors, participants studied, and techniques used, among others (47–49). As a result, scholars agree that the phenomenon may adhere to certain behavioral patterns, such as the election of written text as the most used resource for disseminating inaccurate information (11, 22, 47–49). In addition, the authors agree that vaccine disinformation varies according to particularities in each context studied (11, 50).

On the other hand, scholars have found no consensus regarding the primary social media platform used, the main figure in information disorder, and other variables to consider. Firstly, concerning the leading platform for disseminating information disorder, findings have shown diverse results among Facebook (11, 51), WhatsApp (52), and multiple social media platforms (47, 48). Additionally, scholars have observed that the main social platform varies depending on the country studied (11, 51). Secondly, regarding the leading figures in information disorder, discrepancies have been found between governmental actors (11, 47) and public healthcare actors (49). Thirdly, numerous other variables to consider include analysis levels —including local, national, or global scales—; variable choices for specific targets —such as celebrities, professionals from the public or private sector, government entities, and more—; information format, such as sources used —anonymous, fictional, impersonated, or real—; and topics under discussion —ranging from politics and health to sports and battle conflicts—, among others (52).

## 3 Objective

Since COVID-19 information disorder emerged, vaccine hesitancy has become significantly challenging worldwide (26, 53, 54). In this context, Chile is an attractive case study (55). Historically, this country has been well known for its culture related to high vaccine acceptability, which, in turn, projected high vaccination rates to face the COVID-19 pandemic (56–58). However, before and after the inoculation process, vaccine intention has been lower than expected (12, 59, 60).

Until today, research studies in this country have examined information disorder during the pandemic independently (34, 61) and comparatively with other countries (11, 51, 62, 63). Nevertheless, despite the well-documented prevalence of inaccurate information regarding the COVID-19 vaccination process (64, 65), specific characteristics of this information disorder in Chile remain unexplored. Therefore, this study aims to identify the main characteristics of information disorder that circulated in Chile during the COVID-19 vaccination process, focusing on key topics, actors, and media platforms involved. To achieve this, we address the following research questions:

RQ1: What are the main characteristics of information disorder dealing with fact-checking related to the COVID-19 vaccination process in Chile?

RQ2: What were the main topics surrounding information disorder addressed by fact-checking media during the COVID-19 vaccination process in Chile?

RQ3: Which were the key actors in the information disorder during the COVID-19 vaccination process in Chile?

RQ4: What were the media platforms through which information disorder circulated during the COVID-19 vaccination process in Chile?

## 4 Methods

To answer the research questions, we applied a quantitative research approach using the content analysis technique (66). Based on the fact of comparing the same phenomenon coming from different bodies of texts, we examined articles that belonged to Chilean fact-checking websites: (a) FastCheck.cl, and (b) MalaEspinaCheck.cl, both regarding COVID-19 vaccines. These fact-checking sites were chosen as both media outlets were primary verifiers of viral information related to the pandemic and the only ones that corroborated information disorders pertaining to the COVID-19 vaccination process in Chile. The period explored corresponded from March 2020 to December 2022, considering the period since the COVID-19 pandemic emerged in this country until this paper was produced. The selection criteria applied were based on topics related to the following keywords: vaccines, vaccination, or vaccination process.

### 4.1 Procedures

A total of 226 fact-checking articles were initially obtained. Only those fact-checks classified as containing inaccurate, false, or decontextualized information were selected from them. Therefore, the final sample was composed of a total of 140 texts. Articles that were not fact-checked were not considered in this research, as they do not align with the research questions.

### 4.2 Measures

The selected articles were analyzed based on variables aimed to identify the main characteristics related to false information on vaccination that circulated during COVID-19 in Chile. The operationalization of the variables used to analyze this information disorder was based on previous research during the COVID-19 pandemic (8, 11, 12, 27, 49, 52, 67). The variables considered for this study were operationalized as follows:

- Scope: We analyzed the context of the information regarding its location in three categories: (1) local, (2) national, and (3) international.
- Format: Considering the primary format of each fact-checking article, we categorized its content as mainly: (1) text, (2) audio, (3) video, (4) image, (5) infographic, and (6) more than one format.
- Media Platform: We categorized the media platform used through which information disorder related to vaccine information circulated: (1) WhatsApp, (2) YouTube, (3) Facebook, (4) Instagram, (5) Twitter, (6) others, (7) multiple social media, and (8) traditional media.
- Source: Considering the source of information disorder, we categorized them as (1) fictional, (2) impersonated, (3) real, and (4) anonymous.
- Topics: Based on the literature review, we proposed to classify four topic categories: (1) vaccine side effects, (2) vaccine components, (3) elite power conspiracies, (4) others, and (5) more than one of the categories.
- Key actors: We identified who the main character of the information disorder was distinguishing among: (1) public health actors (individuals or institutions), (2) medical or research professionals (not public actors), (3) political or institutional actors, (4) non-political public actors (individuals or institutions), (5) without a central figure, and (6) collectives.
- Type of Information Disorder: Based on the categorization proposed by Wardle and Derakhshan (33), we identified the primary types of information disorder used: (1) misinformation, (2) disinformation, and (3) malinformation.
- Subject: We categorized the field or area of information disorder as mainly (1) science/health, (2) politics/government, (3) elite/economic/private groups (non-government), and (4) others.
- Type of Strategy: Based on the different types of strategies used to spread information disorder, we categorized them into (1) hoax, (2) exaggeration, (3) decontextualization, and (4) deception.

### 4.3 Data analysis

The extracted data was analyzed according to the variables operationalized by two coders, who used each fact-checking article as their analysis unit. First, we constructed a coding sheet, including all the operationalized variables. Second, to accomplish this task reliably, we calculated inter-coder reliability (ICR) standards (68) from a selected sample of 26 articles, corresponding to 10% of the original sample. As a result, both coders met a coder agreement ranging from 90.6% (Krippendorff = 0.80) to 100% (Krippendorff = 1.0).

Once the coding phase was completed, we performed statistical analysis focusing on the research questions of this study. Research questions 1, 2, 3, and 4 were answered through descriptive statistics analyses. Meanwhile, the Chi-square test was used to examine the association between variables. When the assumptions of the Chi-square test were violated, the Monte Carlo test was used to ensure the accuracy of the results. In the next section, we will present our study results and, later, discuss the implications of our findings.

## 5 Results

During the selected period, from March 2020 to December 2022, the fact-checking articles produced by FastCheck.cl and MalaEspincaCheck.cl were examined. Our initial analysis found that according to each of them: (a) FastCheck.cl produced a total of 1,272 fact-checking articles on several topics (economy, politics, science, health, etc.). Out of these, a total of 273 were related to the COVID-19 pandemic, representing 21.5% of the total; from which, 81 were specifically on the COVID-19 vaccine or vaccination process (6.4% of the total); and (b) MalaEspinaCheck.cl produced 606 articles on different topics from which 136 were related to the COVID-19 pandemic, corresponding to 22.4% of the total; meanwhile only 59 of them addressed specifically COVID-19 vaccine or the vaccination process (9.7% of the total).

### 5.1 RQ1: main characteristics of information disorder related to the COVID-19 vaccination process in Chile

Results of our analysis showed that most of the topics came from international sources, corresponding to 73.6% of the sample; meanwhile, the remaining percentage correlated with Chilean sources.

**Figure 1:**
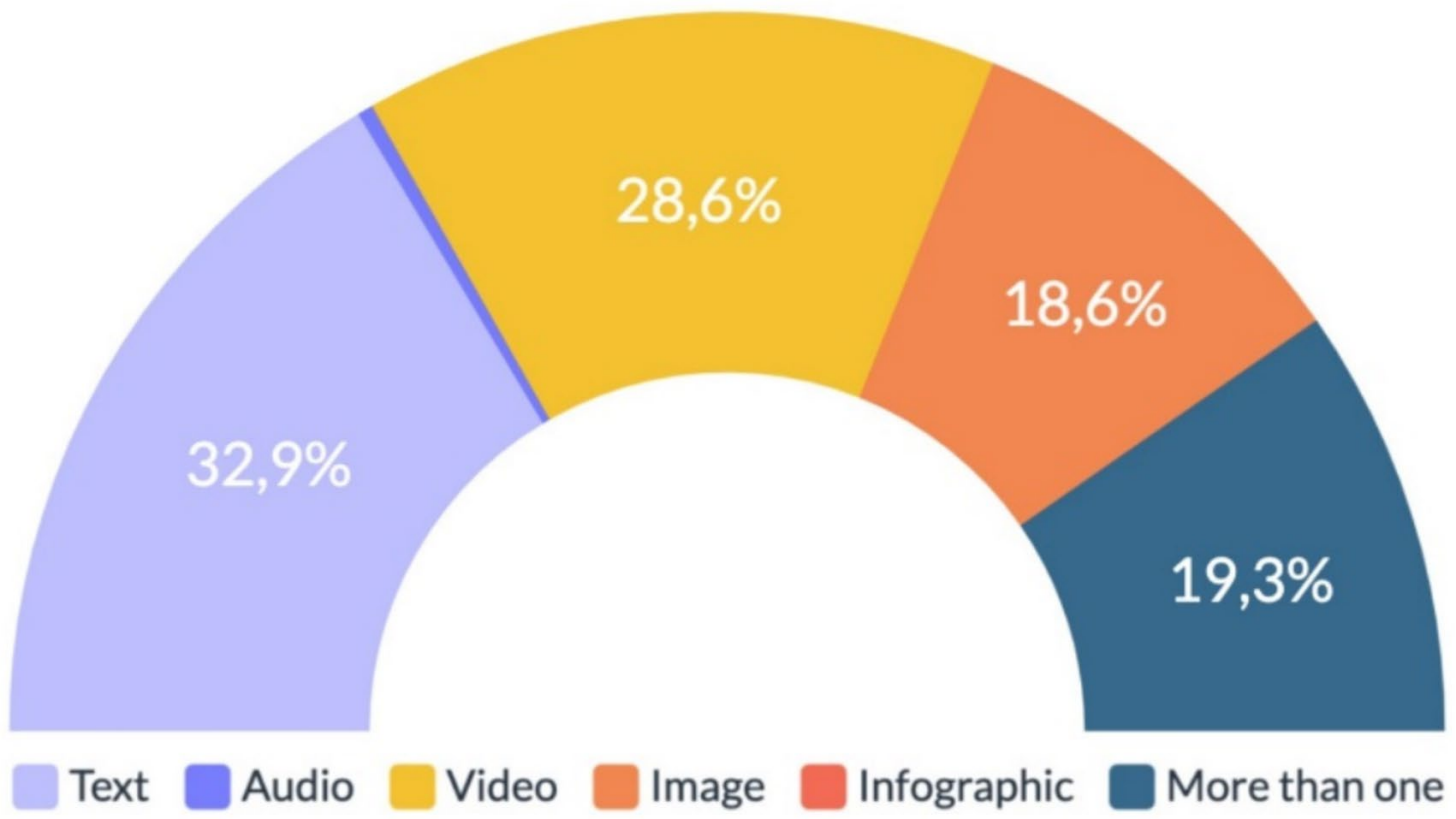
Format used to disseminate information disorder about the vaccination process in Chile. Source: author’s own work.

The most used format for the circulation of informational disorders was text, corresponding to 32.9% (*n* = 49) of the sample, followed by videos (28.6%, *n* = 40) and photography (18.6%, *n* = 26). More than one format for the diffusion of inaccurate information corresponded to 19.3% (*n* = 27) of the cases related to the vaccine and/or the vaccination process against COVID-19. More details are presented in Figure 1.

Concerning the frequency distribution of sources that disseminate inaccurate information, we found out that: (a) 62.9% (*n* = 88) of the sources corresponded to real people who did or said what was stated; (b) 25.7% (*n* = 36) of the cases were impersonated sources and, finally, (c) 11.4% (*n* = 16) of the sources used were anonymous.

No cases of malinformation were registered from the total sample of 140 verifications carried out by Fast Check and Mala Espina. On the contrary, 91.4% (*n* = 128) of the rectified notes responded to disinformation cases; the remaining cases belonged to misinformation. To illustrate this finding, in Table 1, we present the most representative statements that were found in the fact-checking articles studied.

**Table 1:** Examples of types of information disorders identified in this study.

Type of information disorder	Topics	Examples
Disinformation	Vaccine side effects	The 30% of those vaccinated will die within a few months. By court order, a pharmaceutical company had to acknowledge that 82 to 97% of pregnant women who were inoculated aborted their baby.
	Vaccine components	A pharmaceutical company vaccine is produced using cells from aborted fetuses. They have introduced graphene into our bodies.
	Elite power conspiracies	Breaking News! A document has emerged in which a pharmaceutical company gives 2.8 million to the FDA to expedite the approval of their COVID vaccine! Irrefutably exposed bribery! The World Health Organization (WHO) has stated that the COVID-19 pandemic would end in a few months without the need for vaccination. #plandemic
Misinformation	Vaccine side effects	−30 people fainted after receiving doses of the vaccine from a pharmaceutical company in Las Condes. Changes in menstruation and breast issues? Other effects of the COVID-19 vaccine.
	vaccine components	Graphene is one of the components of the vaccine.
Malinformation	No data	No data was found.

Source: author’s own work.

Regarding the subject of information disorder, 70% (*n* = 98) of the articles studied corresponded to the field of science and health, which included topics related to vaccination and/or people’s health. In contrast, 17.8% (*n* = 25) of the cases were on politics and/or government —such as political parties, their members, and governmental matters—; meanwhile, the remaining 9.3% (*n* = 13) and 2.9% (*n* = 4) corresponded to inaccurate information linked to elite groups —which considered individuals or groups of people (companies) associated with economic and private power but not from the public sector—, and other areas, respectively.

**Figure 2:**
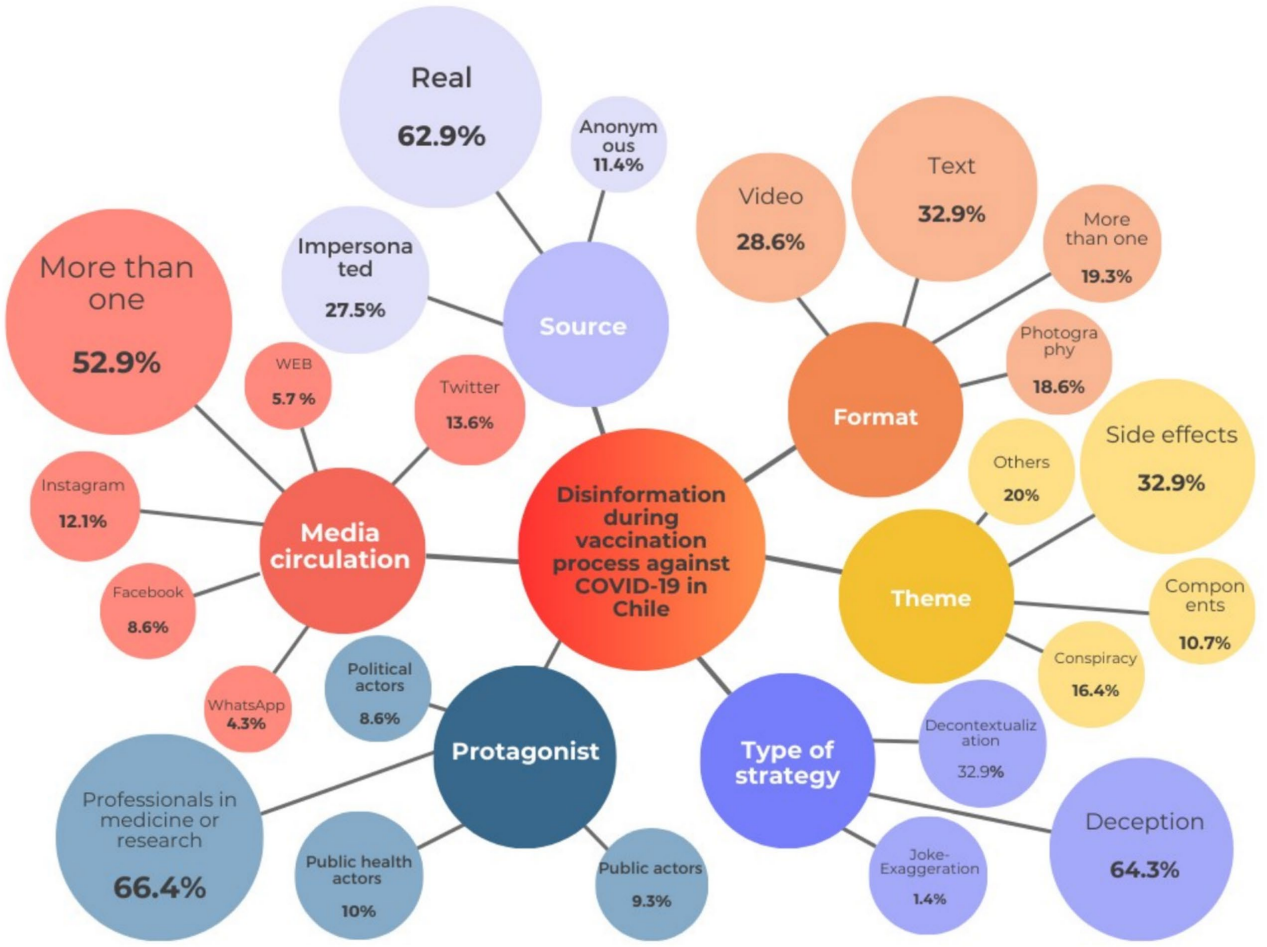
Main characteristics of information disorder related to the COVID-19 vaccination process in Chile. Source: author’s own work.

Finally, the most used information disorder strategy was (a) deception, accounting for 64.3% (*n* = 90), and (b) decontextualization, corresponding to 32.9% (*n* = 46) of the sample. Pranks and exaggerations accounted for 1.4% (*n* = 2), and although the literature acknowledges that both techniques can be highly utilized in disinformation contexts, they were scarcely identified in this study. For more information, see Figure 2.

We additionally analyzed the association between the information source variables and the most used information disorder strategy through chi-square tests. As a result, we identified a significant association when a real source was used with the deception strategy (32.9%, *n* = 46), followed by the decontextualization of information (27.9%, *n* = 39). Besides, the supplant and anonymous source information also tended to be more commonly used with the deception technique, accounting for 20.7% (*n* = 27) and 10.7% (*n* = 15), respectively.

### 5.2 RQ2: main topics surrounding information disorder addressed by fact-checking media during the COVID-19 vaccination process in Chile

**Figure 3:**
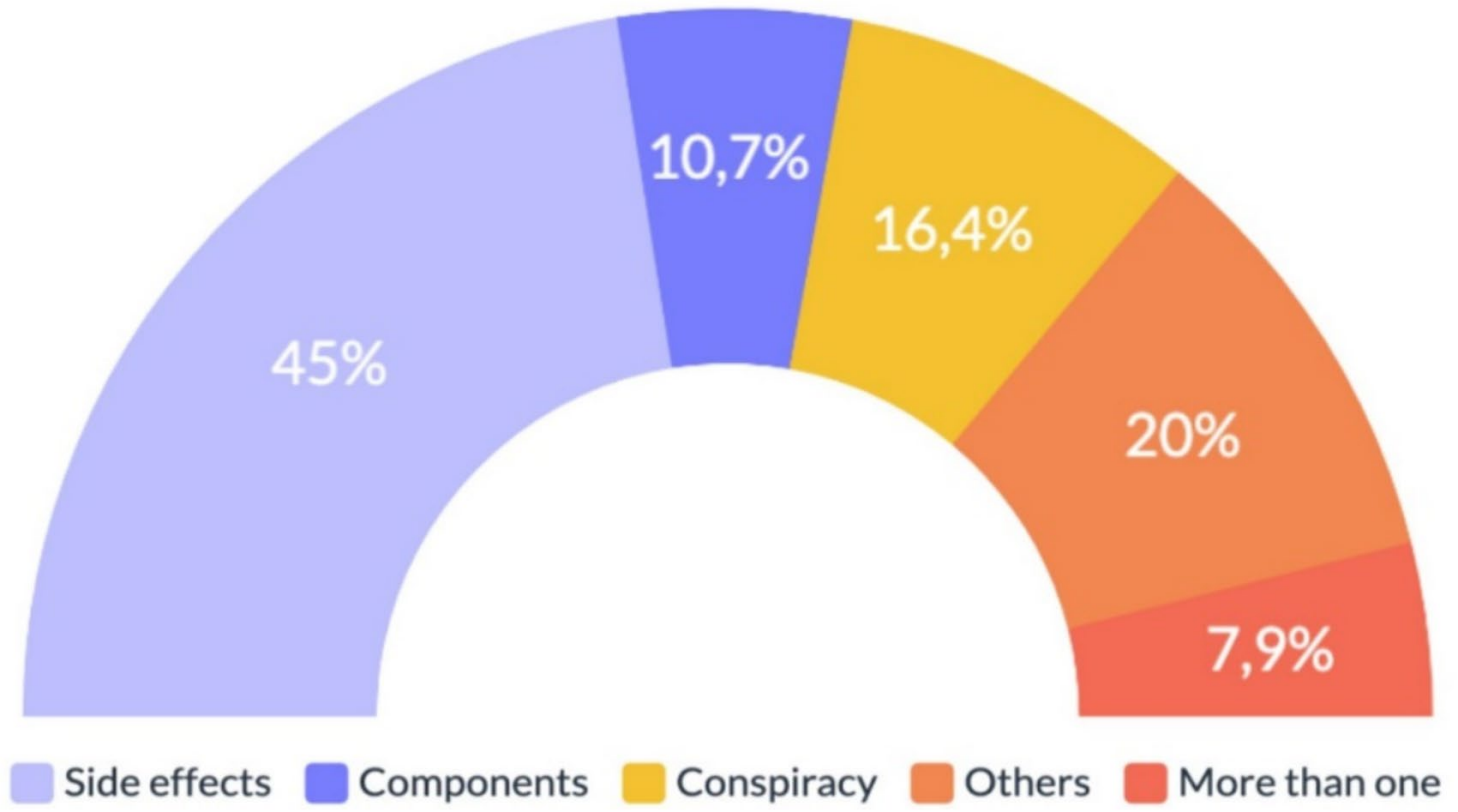
Issues of information disorder in the vaccination process in Chile. Source: author’s own work.

Results showed that 45% (*n* = 63) of fact-checking articles had as main topic vaccination side effects. Mainly, in those articles where scientific evidence was not provided, adverse reactions such as fainting or even death were attributed. Secondly, findings revealed that 20% (*n* = 28) of the articles addressed different topics than those proposed in our variable’s classification. However, these topics were diverse and did not follow a specific trend. Thirdly, 16.4% (*n* = 23) of the articles addressed the topic of conspiracies by power elites. In them, the most recurring themes were affirming that the pandemic and vaccines were a global plan orchestrated by different power groups. In addition, attacks were constantly carried out on pharmaceutical companies and its managers, accusing them of hiding relevant information. Fourthly, 10.7% (*n* = 15) of fact-checking articles focused on the components of the vaccines. In this topic, it was recurrent to find disinformation claiming that vaccines were composed of graphene or aborted fetal cells. Finally, 7.9% (*n* = 11) of the articles studied presented more than one topic. In conclusion, 80% of the articles analyzed were related to the topics of side effects, components, and elite conspiracy, indicating their relevance during the COVID-19 vaccination process. More details are presented in Figure 3.

In addition, a Chi-square test revealed a significant association between the variables topic and the type of technique used. The results indicated that when the main theme of the stories was secondary effects, the most used disinformation technique was deception (25%, *n* = 35), followed by the decontextualization of the information (20%, *n* = 28). The trend was maintained in the other areas, although to a lesser extent. Specifically, when the topic was vaccine components, the deception technique was used (8.6%, *n* = 12), followed by decontextualization (2.1%, *n* = 3); and when the theme was a conspiracy by power elites, the most used technique was deception (11.4%, *n* = 16), followed by decontextualization (4.3%, *n* = 6).

### 5.3 RQ3: key actors in the information disorder during the COVID-19 vaccination process in Chile

**Figure 4:**
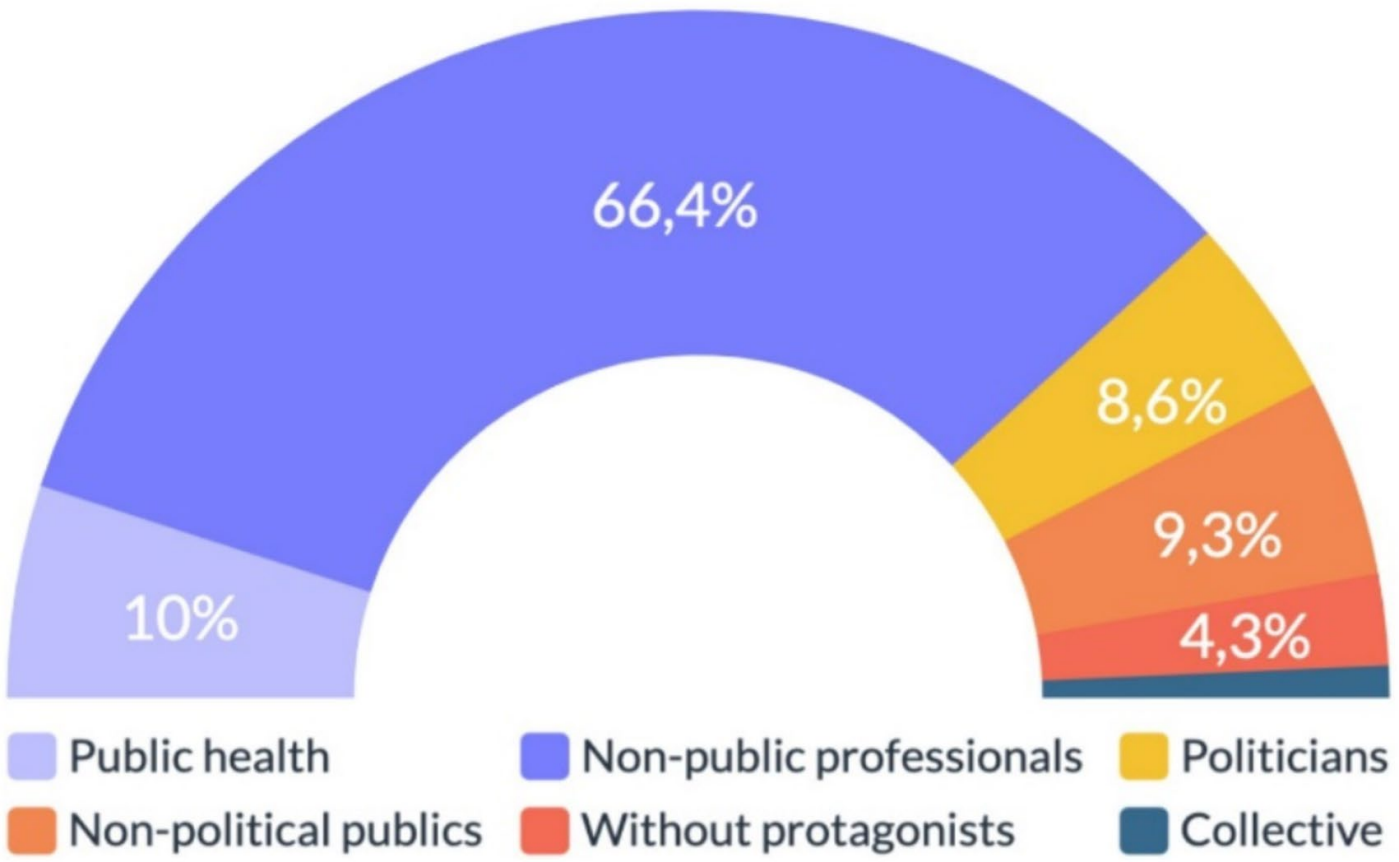
Key actors of information disorder in the vaccination process in Chile. Source: author’s own work.

We found that 66.4% (*n* = 93) of the cases corresponded to non-public health professionals, revealing that key actors were individuals representing a pharmaceutical company or private sector medical professionals. In contrast, individuals coming from the public sector, such as the Ministry of Health or any state institution, accounted only for 10% (*n* = 14) of the total sample. In addition, 9.3% (*n* = 13) of the sample were non-political public figures, such as athletes or actors; meanwhile, 8.6% (*n* = 12) of the time, political actors were the main characters. In 1.4% (*n* = 2) of the cases, the main figure was not an individual person but a collective, and in 4.3%, no protagonist of any kind was detected within the shared story. More details are presented in Figure 4.

In addition, Chi-square tests revealed a statistically significant association between key actors and topic variables, revealing that the most relevant characters disseminated through misinformation were non-public medical professionals, such as researchers, pharmaceutical professionals, or healthcare personnel. These last characters tended to have the greater presence in all the topics addressed as follows: 35% (*n* = 49) in topics related to side effects, 7.9% (*n* = 11) in vaccine components, 8.6% (*n* = 12) in elite conspiracy, 7.9% (*n* = 11) in other topics, and 7.1% (*n* = 10) when more than one of the topics was mentioned.

### 5.4 RQ4: media platform through which information disorder circulated during the COVID-19 vaccination process in Chile

**Figure 5:**
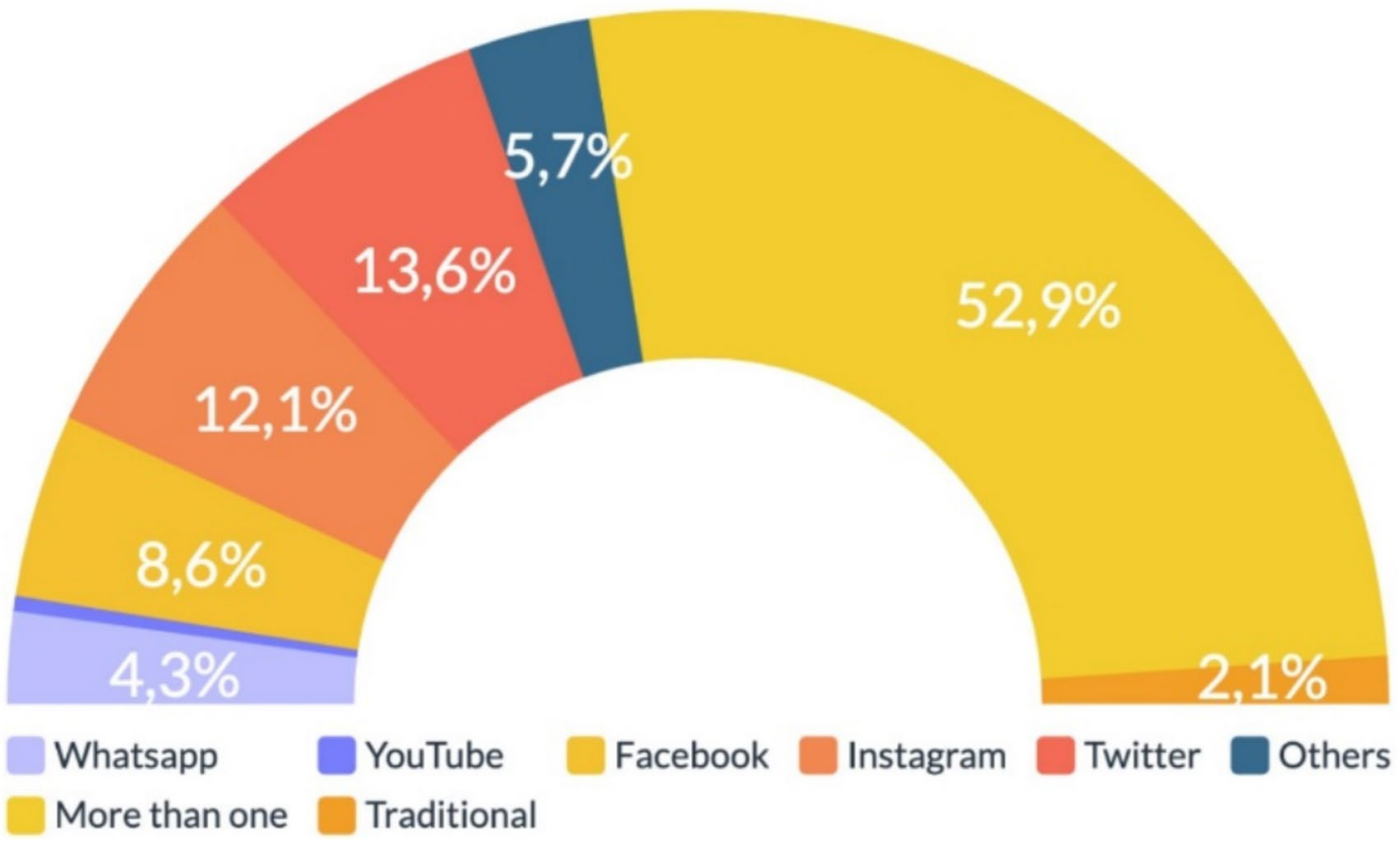
Media platform of information disorder circulation in the vaccination process in Chile. Source: author’s own work.

We identified that 52.9% (*n* = 74) of the information disorder tended to circulate through more than one media outlet, following this trend: 13.6% (*n* = 19) circulated exclusively on Twitter, 12.1% (*n* = 17) on Instagram, 8.6% (*n* = 12) on Facebook, 5.7% (*n* = 8) through other types of media like Telegram or websites, 4.3% (*n* = 6) on WhatsApp, 2.1% (*n* = 3) through traditional media, and only 0.7% (*n* = 1) via YouTube. More details are presented in Figure 5.

## 6 Discussion

This research proposes a novel approach as disinformation disorder about the vaccination process during the COVID-19 pandemic is studied for the first time in Chile. This is important because it provides a foundation for future research related to the vaccination process and the COVID-19 pandemic in a Latin American country that has not yet been studied. In addition, the fact of analyzing information disorder directly from two of the main fact-checking media outlets in Chile —Fast Check and Mala Espina— provided an incommensurable contribution as they belong to the International Fact-Checking Network (IFCN) and are responsible for corroborating inaccurate information related to the COVID-19 vaccination process and the vaccine itself.

Within the public discourse surrounding the COVID-19 vaccine infodemic worldwide, the findings of this study confirm similarities with specialized literature as well as specific characteristics of the information disorder present in Chile. In addressing RQ1, it was observed that text was the most used format for disseminating false information (32.9%, *n* = 49), as noted in previous studies in Spain and Italy (48, 52), this study revealed a more significant prevalence of visual representation. Videos and images together accounted for 47.2% (*n* = 66). This constitutes a novel finding that requires further and more research, as the pivotal role of visuals in information disorder is getting more common each day.

Another relevant finding addressing RQ2 showed that vaccine side effects (45%, *n* = 63) and vaccine components (10.7%, *n* = 15) were fundamental topics presented, both belonging to the area of science/health. These findings follow the trend of other research in Latin America, Spain, and China that has also identified a constant effort to discredit vaccines and the health sector (3, 51, 69). This is particularly significant given that previous studies in the United States and Portugal have found that concerns about COVID-19 vaccine safety were strong predictors of vaccine hesitancy (70, 71). Therefore, the spread of information disorder regarding COVID-19 vaccine safety could negatively impact people’s willingness to vaccinate. Besides this finding, elite power conspiracy constitutes a crucial influence that is present in Chilean information disorder in the vaccination process. The danger of these lies in the credibility they can gain in society because, mainly being based on false or out-of-context information, they appear more believable and can motivate people to share and propagate inaccurate information within their social circles, exacerbating vaccine skepticism or rejection, which, in turn, hindered the fight against the pandemic.

Given this context, the complexity involved in creating and disseminating vaccine-related information disorder highlights the need to implement innovative tools to mitigate its potential harm to public trust and vaccine acceptance. For example, prior research has demonstrated that AI technologies can play a critical role in pandemic management by accelerating diagnostic processes and supporting risk assessment (16, 21, 72). Although these studies primarily focus on the clinical and epidemiological aspects of COVID-19, the reasoning behind using AI for rapid data processing and decision-making also applies to the early detection and containment of information disorder. These tools present promising opportunities for supporting evidence-based communication strategies and reducing the spread of inaccurate information during health emergencies.

Regarding RQ3, our results revealed that 66.4% (*n* = 93) of the cases corresponded to non-public health professionals, such as medical or research professionals. This specific finding differs from the literature review in which scholars had proven that during the pandemic, governmental actors (11, 47) and public healthcare actors (49) were the main figures of information disorder. We believe that this disparate result is explained by the fact that the subject of science/health was the most prominent area that emerged from our analysis. Vaccines and their side effects correspond to the topic addressed mainly by health professionals belonging to pharmaceutical companies or private health organizations that were not well known by the Chilean public. Therefore, these key actors were not considered public figures, as clearly identified by most of the Chilean people. Nevertheless, this last hypothesis requires further investigation in future studies.

Finally, analyzing the primary media platforms through which inaccurate information circulated in Chile (RQ4), it is important to note the complexity of how any form of information disorder spreads. Specifically, in contrast to other countries (11, 51), in Chile, the same piece of information disorder tends to be repeated across various media simultaneously —Twitter, Facebook, Instagram, and WhatsApp—. This follows the trend observed by authors such as López-Martín and Córdoba-Cabús (73) and Skafle et al., (74) in Europe and USA. One of the reasons that we hold for this alarming result is that it makes it challenging to identify the origin or party responsible for the information disorder. This last issue intensifies the impact of information disorder as social media platforms have exacerbated this problem due to its immediacy. According to Morejón-Llamas (51), in the Latin American region, information disorder can take between 2.2 and 67 days to be verified and debunked by fact-checking organizations, which corresponds to a crucial time in which information disorder can or may have a significant impact on societies well-being. In addition, given the fact that primary media platforms for information disorder circulation are similar in Chile and other Latin American countries, it would be helpful to propose joint regulatory strategies to address this issue. As suggested by researchers in other contexts, such as Europe, collaborative approaches may help address this issue (75–79).

## 7 Conclusion

This study identifies the main characteristics of the information disorder circulated in Chile during the COVID-19 vaccination process. It is important to highlight that this study primarily focused on science/health. At the same time, the theoretical framework of information disorder usually considers general topics and does not reach specific themes such as vaccination processes. Also, in the Chilean case, it is relevant to notice the lack of fact-checking processes (67). However, FastCheck.cl and MalaEspinaCheck.cl do relevant work, as they also belong to the International Fact-Checking Network. Nevertheless, Chile is ranked as one of the countries with the lowest number of fact-checks related to COVID-19 and the vaccination process (51, 80), health communication scholars need to understand the importance of fact-checking health information disorders, specifically related to vaccination (35, 75) due the potential influence that information disorder can have on vaccination processes.

### 7.1 Limitations

Firstly, the selected sample is small, reflecting the low number of verifications conducted in the country during the studied period. Therefore, it would be interesting to reply to this same research in other Latin American countries with more vaccine disorder information to study. And secondly, this work focused only on quantitative research areas. Therefore, it is also necessary to examine the qualitative context or frames in which vaccination processes, as in the Chilean case, were lived. Thus, this research needs to be deepened by other health communication scholars.

## Data Availability

The original contributions presented in the study are included in the article/supplementary materials, further inquiries can be directed to the corresponding author.
